# Systematic review of surgical treatment techniques for adult and pediatric patients with pectus excavatum

**DOI:** 10.1186/1749-8090-9-25

**Published:** 2014-02-07

**Authors:** William Rainey Johnson, David Fedor, Sunil Singhal

**Affiliations:** 1 Department of Surgery, Thoracic Surgery Research Laboratory, Perelman School of Medicine, Philadelphia, PA, USA

**Keywords:** Pectus excavatum, Pediatrics, Adults, Nuss procedure, Ravitch procedure

## Abstract

This compares outcome measures of current pectus excavatum (PEx) treatments, namely the Nuss and Ravitch procedures, in pediatric and adult patients. Original investigations that stratified PEx patients based on current treatment and age (pediatric = 0–21; adult 17–99) were considered for inclusion. Outcome measures were: operation duration, analgesia duration, blood loss, length of stay (LOS), outcome ratings, complications, and percentage requiring reoperations. Adult implant patients (18.8%) had higher reoperation rates than adult Nuss or Ravitch patients (5.3% and 3.3% respectively). Adult Nuss patients had longer LOS (7.3 days), more strut/bar displacement (6.1%), and more epidural analgesia (3 days) than adult Ravitch patients (2.9 days, 0%, 0 days). Excluding pectus bar and strut displacements, pediatric and adult Nuss patients tended to have higher complication rates (pediatric - 38%; adult - 21%) compared to pediatric and adult Ravitch patients (12.5%; 8%). Pediatric Ravitch patients clearly had more strut displacements than adult Ravitch patients (0% and 6.4% respectively). These results suggest significantly better results in common PEx surgical repair techniques (i.e. Nuss and Ravitch) than uncommon techniques (i.e. Implants and Robicsek). The results suggest slightly better outcomes in pediatric Nuss procedure patients as compared with all other groups. We recommend that symptomatic pediatric patients with uncomplicated PEx receive the Nuss procedure. We suggest that adult patients receive the Nuss or Ravitch procedure, even though the long-term complication rates of the adult Nuss procedure require more investigation.

## Introduction

Pectus excavatum (PEx), or “funnel chest,” is the most common congenital chest wall abnormality, affecting roughly 1:400 live births [1,2]. Pectus excavatum affects males four times as often as females, typically presenting in early childhood [3]. The etiology remains unclear, but it appears to be polygenetic, following autosomal dominant, autosomal recessive, X-linked, or sporadic patterns of inheritance [2-4]. The defect is thought to result from unbalanced growth of the costochondral regions of the anterior chest wall, leading to symmetric and asymmetric anomalies [5]. Regardless of etiology, PEx has an adverse effect on many patients’ lives.

Symptom severity varies from completely asymptomatic to clinically psychologically debilitating and symptomatic. The most common symptoms include dyspnea (especially with exercise), exercise intolerance, and chest pain [6]. Patients often have body image embarrassment, which may result in adverse psychological symptoms and lower quality-of-life [7-9]. While PEx is often considered a purely cosmetic disorder, Kelly and colleagues reviewed autopsies and concluded that patients with PEx have a shorter life expectancy [10]. Furthermore, severity of pectus excavatum is associated with reduced pulmonary function [11,12].

Bauhinus first described PEx in the 16th century, but the first breakthrough in management came in 1949 when Ravitch described costochondral osteotomy to repair PEx [13,14]. Sixty years later the Ravitch procedure remains a common PEx treatment, albeit in a highly modified form [5,13]. Robicsek and colleagues made modifications to the open procedure in the 1960s, using sternal turnover and stabilizing mesh, which continues to be reported today [15,16]. Also in the 1960s cosmetic interventions became available for asymptomatic PEx patients [17]. Today, silicon implants and polyethylene implants are manipulated to cosmetically fix the deformity [18-20]. In 1998, Nuss introduced a minimally invasive repair, temporarily implanting metal bars to alter the curvature of the anterior chest wall. This has gained popularity throughout the past decade [21]. Amidst the mass of literature and propaganda, it can be challenging for patients and physicians to select the most appropriate treatment. A handful of studies compare the two most common therapies – Ravitch and Nuss procedures [22-31]. However, the relationship of age to these therapies has not been well examined. Kim and colleagues and Ohno and colleagues provided some evidence to address the age appropriateness of the minimally invasive Nuss procedure, but their data were not sufficient to answer the question for all treatment modalities and were derived from small cohorts (n < 30) [32,33]. This systematic analysis provides a collection, synthesis, and analysis of all relevant literature published since 1949 in order to objectively evaluate and compare the outcomes of different PEx treatment modalities in children and adults.

## Methods

PubMed was searched for articles published in English between January 1949 and July 2012. Search terms included “Pectus Excavatum Treatment” (1366 results), “Nuss Procedure” (421 results), “Ravitch Pectus Excavatum” (130 results), “Open Pectus Excavatum” (81 results), “Mesh Pectus Excavatum” (17 results), “Vacuum Pectus Excavatum” (2 results), “Orthotic Pectus Excavatum” (5 results), “Physiotherapy Pectus Excavatum” (31 results), “Pectus Excavatum Psychological Therapy” (47 results), “Non-surgical pectus excavatum treatment” (1 result), and “conservative pectus excavatum treatment” (20 results). One investigator (WRJ) performed all the searching and filtering in order to maintain selection continuity. Based on the abstracts and titles, studies that grouped patients by treatment type, focused on PEx, contained >80% primary patients, and included data-categorization on patients’ ages (Pediatric: 0 – 21; Adult 17 – 99) were included. We anticipated a consistent cut-off close to 19 years old because the force required to elevate the sternum significantly increases at this age due to developmental changes [34]. Variation in age categorization among some of the larger studies precluded our ability to have a discrete cut-off between adults and pediatric patients, requiring us to accept overlapping age ranges. When the abstract and title did not contain sufficient information to answer our initial inclusion criteria, the study was included in the next round of filtering (full-text review). After duplicates were discarded, 135 full-text manuscripts were reviewed.

**Figure 1:**
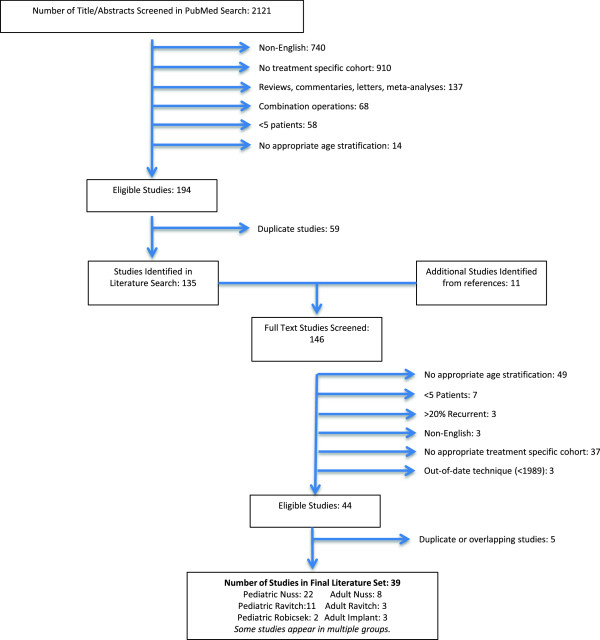
This shows the selection and filtration process.

The methods, results, and references were reviewed for each of these articles. Those that met the initial criteria: contained more than 5 patients, less than 20% recurrent patients, patients with an average Haller or equivalent index greater than 3.2; and used a currently practiced technique were included. If multiple articles had overlapping cohorts (determined by institution and year), only the most recent publication was included. In reviewing the references, articles that had not previously been filtered were added to the selection process (n = 11). Finally, publications that described surgical techniques that differ significantly from current techniques were excluded; in practice, this removed all publication prior to 1989. Forty-four articles met the criteria for inclusion. These were stratified based on age range and treatment, providing seven subgroups: Nuss adult (n = 8) [32,35-41], Nuss pediatric (n = 22) [21,23-25,27,29,30,32,33,35],[39,42-52], Ravitch adult (n = 3) [53-55], Ravitch pediatric (n = 11) [24,25,27,29,30,55-60], Robicsek pediatric (n = 2) [57,61], Implant adult (n = 3) [18,20,62]. No other treatments had articles that satisfied the inclusion criteria. Figure 1 diagrams our selection and filtration process.

The data from each study was harvested for: study type, number of patients, age, follow-up duration, operation duration, analgesia duration (epidural and intravenous), blood loss, post-operative length of stay (LOS), percentage good/excellent outcome ratings (as determined using Humphreys’ and Jaretzki’s model [63]), percentage of non-displacement complications (i.e. complications that did not involve bar or strut displacement), self-resolving complications (e.g. mild atelectasis), percentage requiring reoperation (i.e. patients that recurred and/or required reoperation), percentage bar/strut displacement, and percentage bars/struts removed. Note that pectus bars are used in Nuss procedures and substernal struts are used in some Ravitch procedures. Recognizing the follow-up time dependence of bar/strut displacement as a complication we separately examined non-displacement complication rates. Trinary variables (better, same, worse) were used to assess preoperative and postoperative symptoms, lung function, and cardiac function. The designation ‘better,’ ‘same,’ or ‘worse’ was assigned based on criteria of authors of each particular study. All studies that reported >20% scoliosis patients and >3% Marfans patients, indicating a significant deviation from the average population were noted (Tables 1, 2 and 3). All averages were weighted based on study size (Table 4). These weighted averages were calculated for each quantifiable variable to facilitate group comparisons. Only reported means were included in the calculations; medians were excluded. We categorized quantifiable outcomes comparisons as showing a: (1) clear difference, (2) slight difference, (3) tendency, and (4) similarity. A ‘clear difference’ was denoted when the ranges did not overlap at all and the averages each fall outside of the comparison group’s range. Clear differences were statistically different. A ‘slight difference’ was noted when the ranges overlapped marginally and the averages each fell outside of the comparison group’s range. Slight differences were likely statistically significant. A ‘tendency’ was noted when the ranges were mostly overlapping and the averages, while fitting within the comparison group’s range, demonstrated clusters of data points at opposite ends of the range. Tendencies had a low probability of being statistically significant. Finally, a ‘similarity’ was noted when ranges were almost entirely overlapping and averages were clustered in the same half of the range (i.e. overlapping ranges and averages). Similarities were statistically the same. If a range was not available, it was assumed to overlap. If an average was not available, no comparison was made.

**Table 1:** This is a comprehensive list of all the studies that included the Nuss procedure

**Reference**	**n**	**Study type**	**Follow-up (years)**	**Age, mean (range), years**	**Operation duration, min**	**Analgesia duration, epidural/ IV, days**	**Mean blood loss, mL**	**Mean LOS, days**	**Outcome, >good/ excellent, %**	**Non-displacement complication/ self-resolving, %**	**Requiring reoperation, %**	**Bar displacement, %**	**Number of bars removed, %**	**Symptoms**	**Lung function**	**Cardiac function**
**Esteves [**[35]**]**^**a**^	**19**	**R**		**22.6 (20–27)**	**96.5**	**3/--**		**6.1**		**0/--**			**21**			
**Teh [**[36]**]**	**19**	**R**	**2.4 (.3-5.9)**	**19.5 (17–42)**	**126**	**3/--**		**5.8**		**42/31**	**10.5**	**10.5**	**32**			
**Cheng [**[37]**]**^**b**^	**96**	**P**	**1.8 (0.4 - 3.0)**	**24.5 (18–42)**	**80**^**c**^		**<10**	**7.2**	**92/73**	**3/1**	**2**	**2**	**7**			
**Hebra [**[38]**]**	**30**	**R**		**23 (18–32)**		**3/--**		**6**	**86/50**	**20/4**		**6.7**				
**Aronson [**[39]**]**	**35**			**23 (18–47)**	**65**^**c**^	**(4–5)/--**		**7**^**c**^		**34/--**		**14.3**	**40**			
**Schalamon [**[40]**]**^**b**^	**43**	**P**	**1.9 (0.5 - 5)**	**22 (18–39)**	**70**			**9.3**	**--/91**	**23/--**	**0**	**2.3**	**35**		**Same**	
**Kim [**[32]**]**	**12**	**R**	**3.4**	**27 (20–52)**	**127.3**			**10**	**64/--**	**58/--**	**42**	**8.3**				
**Coln [**[41]**]**	**8**	**R**	**1.8**	**(19–32)**	**92**	**2.8/<5**		**4**		**100/50**	**0**	**25**	**50**	**Better**		**Better**
Zganjer [42]	128	R	3.6	13.8 (8–21)		--/4	35	10	95/73^d^	41/20	4.7	4.7	42	Better		Better
Esteves [35]^a^	26	R		15.3 (5.0-19)	90.3	3/--		5.07		4/--			12			
Nuri [43]	12	R	3.6 (0.3 - 7)	9 (4.0-21)							0	33.3				
Densmore [44]	117	R	4	12.9 (8.0-18)	118	--/3.8	<10	5.8		21/--	16	12	100			
Mao [45]	115	R		7.9 (2.7-18)	59.5	--/5.1		8.5	98/90	20/6	1.7	2.6	32			
Felts [46]	25		2.2	13.8 (5.0-18)		3/--		7	96/--	20/--	4	4	52		Better	
Lam [25]	19	R		15.4 (13–18)	72.1	3.7/--		4.5								
Sigalet [47]	26	P	>2	13.2				5			11.5	11.5		Better	Better	Same
Kubiak [48]	15			15.9 (10.7-18.1)						33/20	6.7	13.3			Better	Better
Kelly [24]^a,b^	284	P	0.2	13.6 (8.0-21)					97/90	98/47		2.5				
Coln [49]^a^	123	R	<2	13 (5.0-18)		--/2.9		3.1		11/--		0.8		Better		Better
Aronson [39]	141			13 (5.0-17)	65^c^			7^c^		24/--	0.7	12.8	55			
Kim [32]	39	R	3.4	8.9 (1.5-19)	60.7			5.9	83/--	25/--	7.7	5.1				
Bohosiewicz [50]	66	R		11.8 (1.0-19)					--/85	15/--	1.5		36			
Watanabe [51]	53	R		9 (4.0-18)	76	--/4.3	4	8.9		26/2	3.8	7.5				
Ohno [33]	23	R		7.6 (3.0-19)	143.5		16.5	7.9	--/78	13/--		8.7				
Inge [30]	43	R	1.4 (0.6 - 2.8)	11 (4–19)	70	0/--		2.4	90/	14/--	4.7	4.7				
Fonkalsrud [34]	68	R		12 (5.0-19)	75	3/5	90	6.5		13/--	10.2	8.8	26			
Molik [27]	35	R		9.5 (5.0-20)	198	3/3.8		4.8		37/--	22.9	17.1				
Miller [29]	80	R		9.4 (<21)	53		20	3.7	95/--	45/8	5	5	20	Better		
Engum [52]	20	R	1.2 (0.2-1.6)	8.2 (5–15)				4.9		40/35	15	20				
Nuss [21]	42	R	4.6 (1–9.2)	(<15)			15	4.3	87/73^d^	24/7	4.8	4.8				

The table is divided horizontally with bold & plain text to differentiate between pediatric and adult patients. Within each subgroup studies are organized in reverse chronological order from top to bottom based on publication date.

^a^= >3% patients reported to have Marfans.

^b^ = >20% of patients reported to have scoliosis.

^c^ = Median (not included in weighted averages).

^d^ = specified as long-term outcomes.

P/R = Prospective/Retrospective Cohort Study.

**Table 2:** This is a comprehensive list of all the studies that included the Ravitch procedure

**Reference**	**n**	**Study type**	**Follow-up (years)**	**Age, mean (range), years**	**Operation duration, min**	**Analgesia duration, epidural/ IV, days**	**Mean blood loss, mL**	**LOS, days**	**Outcome, >good/ excellent, %**	**Non-displacement complication, attention/ self-resolving, %**	**Requiring reoperation, %**	**Strut displacement, %**	**Number of struts removed, %**	**Symptoms**	**Lung function**	**Cardiac function**
**Neviere [**[53]**]**	**70**	**P**	**1**	**27 (18–62)**											**Better**	**Better**
**Jaroszewski [**[54]**]**^**b**^	**320**	**R**	**2.2; (0.1 - 21)**	**27 (19–67)**	**191**	**0/2**		**2.9**		**8/--**	**1**^**d**^	**0**		**Better**		
**Haller [**[55]**]**	**108**	**R**		**(17–39)**					**91/60**		**10**					
Lam [25]	24			15.5 (13–18)	84.1	1.3/2.8		3.9				50				
Hu [56]	398	R	4.2 (1–16)	4.6 (2.5-18)					99/--	1.3/--	7	1		Better	Better	Better
Kelly [24]^a,b^	43	P	0.2	15.9 (8.0-21)					98/68	63/42				Better		
Inge [30]	25	R	3.5 (2.5-4)	12 (4–18)	195	0/--	197	4.4	85/--	4/--	0	0				
Lansman [57]	75	R	0.5-13	8.2 (<16)	121					41/--	17	17.3				
Molik [27]	68	R		12.6 (5.0-20)	282	0.6/1.8		4		19.1/--	6	0				
Miller [29]	32	R		11.5 (0–21)	143		200	3.2	94/--	18.8/--	3			Better		
Lane-Smith [58]	161	R	8.8 (1–21.5)	6.4 (2–17)	150		80	6.1^c^	83/--		7					
Haller [55]	352	R	>2	(1–17)					99/78		<1					
Gilbert [59]	32	R	2.2	(3–16)						3.1/3.1	0	34	100			
Holcomb [60]	40	R	3.8	(2–16)					95/68		5					

The table is divided horizontally with bold and plain text to differentiate between pediatric and adult patients. Within each subgroup studies are organized in reverse chronological order from top to bottom based on publication date.

^a^ = >3% patients reported to have marfans.

^b^ = >20% of patients reported to have scoliosis.

^c^ = decreased to 4.8 after 1986.

^d^ = occurred before minimal cartilage resection.

P/R = Prospective/Retrospective Cohort Study.

**Table 3:** This is a comprehensive list of all the studies that included Robicsek and Implant procedures

**Reference**	**n**	**Study type**	**Follow-up (years)**	**Age, mean (range), years**	**Operation duration range, min**	**Anasthesia duration, Epi/IV, days**	**Mean blood loss, mL**	**Mean LOS, days**	**Outcome, >good/ excellent, %**	**Non-displacement complication, attention/ self-resolving, %**	**Requiring reoperation, %**	**Bar/strut displacement, %**	**Number of bars removed, %**	**Symptoms**	**Lung function**	**Cardiac function improved**
**Snel [**[20]**]**	**16**	**R**	**6 (0.5-20)**	**43 (21–64)**					**69/6**	**62.5/--**	**18.8**					
**Grappolini [**[18]**]**	**11**		**16.3**	**29 (21–47)**	**137.2**			**3.6**		**0/--**						
**Wechselberger [**[62]**]**	**20**	**R**	**4.5 (0.8-11.5)**	**20 (17–42)**				**5**	**90/80**	**85/--**						
Luzzi [61]	23	R		16				5	89/64	47.8/--	9			Better	No change	
Lansman [57]	8	R	(0.5-13)	<16	87	0/--			88/38	12.5/--	0	12.5				

The table is divided horizontally with bold and plain text to differentiate between pediatric Robicsek and adult surgical implant patients. Within each subgroup studies are organized in reverse chronological order from top to bottom based on publication date.

P/R = Prospective/Retrospective Cohort Study.

**Table 4:** This displays the weighted averages based on cohort size for each of our subgroups

**Cohort**	**Ravitch adult**	**Nuss adult**	**Implant adult**	**Robicsek pediatric**	**Ravitch pediatric**	**Nuss pediatric**
**Number of references**	**3**	**8**	**3**	2	13	22
**Follow-up, mean (range) years**	**2.0 (0.08 - 21) (n = 390)**	**2 (0.3 - 5.9) (n = 178)**	**7.8 (n = 47)**		3.9 (0.2 - 21.5) (n = 1096)	2.1 (0.2-7) (n = 859)
**Age, mean (range), years**	**27 (18–67) (n = 390)**	**23.3 (17–52) (n = 254)**	**29.9 (21–64) (n = 47)**		7.2 (0–21) (n = 809)	12.0 (1.5-21, n = 1458)
**Operation duration mean (range), min**	**191 (165–217, n = 320)**	**94 (65–128, n = 101)**	**137.2 (n = 11)**	87 (n = 8)	166 (84–282, n = 385)	86 (65–198, n = 618)
**Anasthesia duration, Epi/IV, days**	**0/2 (n = 320)**	**3 (2.8-3, n = 76)**		0 (n = 8)/	0.6 (0–1.3, n = 117)/2.1 (1.8-20, n = 92)	2.5 (0–3, n = 216)/4.1 (3–5.1, n = 639)
**Mean blood loss, mL**		**<10 (n = 96)**			111 (80–200, n = 218)	28.6 (4–90, n = 511)
**Mean length of stay, days**	**2.9 (n = 320)**	**7.3 (4–10, n = 227)**	**4.5 (3.6-5, n = 31)**	5 (n = 23)	5 (3.2-6.1, n = 310)	6.1 (3.1-10, n = 982)
**Outcome, >good/excellent, %**	**91/60 (n = 108)**	**88 (64–92, n = 138)/73 (50–91, n = 168)**	**81 (69–90, n = 36) /47 (6–80, n = 36)**	89 (88–89, n = 31)/54 (38–64, n = 31)	96 (83–99, n = 1051)/76 (68–78, n = 435)	95 (83–97, n = 756)/85 (73–90, n = 658)
**Non-displacement complication/self-resolving, %**	**8 (n = 320)/**	**21 (0–100, n = 262)/8 (1–50, n = 153)**	**57.4 (0–85, n = 36)/**	36 (12.5-47.8, n = 31)/	12.5 (1.3-63, n = 673)/25 (3.1-42, n = 75)	38 (4–98, n = 1443)/25 (2–47, n = 737)
**Requiring reoperation, %**	**3.3 (1–10, n = 428)**	**5.3 (0–42, n = 178)**	**18.8 (n = 16)**	6.3 (0–9, n = 31)	5.3 (0–17, n = 1183)	6.3 (0–23, n = 1025)
**Bar/strut displacement, %**	**0 (n = 320)**	**6.1 (2–25, n = 243)**		12.5 (n = 8)	6.4 (0–50, n = 622)	6.6 (0.8-33.3, n = 1389)
**Number of bars removed, %**		**23 (n = 220)**			100 (n = 32)	47 (n = 766)
**Symptoms**	**Better (n = 320)**	**Better (n = 8)**		Better (n = 23)	Better (n = 473)	Better (n = 357)
**Lung function**	**Better (n = 70)**	**Same (n = 43)**		No change (n = 23)	Better (n = 398)	Better (n = 66)
**Cardiac function improved**	**Better (n = 70)**	**Better (n = 8)**			Better (n = 398)	Better (n = 292)

The table is divided vertically with bold and plain text to differentiate between pediatric and adult patients.

## Results

### Comparison of adult groups

Two hundred and sixty-two adult minimally invasive Nuss procedure patients were presented in eight different studies (Table 1) [21,64]. Four hundred and ninety-eight adult open, highly modified Ravitch procedure patients were presented in three different studies (Table 2) [5]. Forty-seven adult surgical implant patients appeared in 3 different investigations (Table 3). The patients were similar in terms of age and incidence of secondary operation because of our selection criteria.

The outcome ratings between the Nuss and Ravitch procedures were similar (Table 4). In addition, there was at least one study in each of the Nuss and Ravitch groups that found significant improvements in postoperative symptoms compared to preoperative symptoms [41,53,54]. There was no report of surgical implants improving clinical symptoms (e.g. dyspnea, exercise intolerance, lung function, etc.).

These three treatments differed in many respects (Table 4). There was a slight difference in operation time; the Ravitch procedure took longer (191 min) than the Nuss procedure (94 min). The surgical implants took an average of 137.2 min; however, a larger portion of these required reoperation (mostly due to implant displacement). There was a clear difference in the use of epidurals; none of the Ravitch patients received epidurals, while Nuss patients averaged 3 days of epidural use. There was a slight difference in the LOS; 7.3 days for Nuss patients versus 2.9 days for Ravtich patients. The surgical implant patients (4.5 days) showed a tendency to be hospitalized for shorter time than the Nuss patients, but were similar to Ravitch patients.

The complication rates varied greatly among studies, but ranges of all three procedures overlapped. The average non-displacement complication rate for the Ravitch procedure (8%) was much lower than the other two procedures (Nuss 21%, Implant 57.4%), suggesting a tendency for fewer clinically relevant complications with the Ravitch procedure. Inclusive of strut and pectus bar displacements, the Ravitch complication rate did not increase, but the Nuss procedure complication rate increased to 27.1%. Notably, however, many of the Nuss procedure complications were minor and self-resolving pneumothoraxes or atelectasis (8%); whereas, many of the implant complications were seromas, infections, and implant displacements. Finally, the percentages requiring reoperation were similar for the Nuss (5.3%) and Ravitch (3.3%) procedures, but the implants procedure had a tendency to have higher percentage requiring reoperation (18.8%).

### Comparison of pediatric groups

We identified 1500 pediatric Nuss procedure patients in 22 investigations, 1186 pediatric highly modified Ravitch procedure patients in 10 studies, and 31 pediatric Robicsek procedure patients in 2 studies (Tables 1, 2 and 3). These studies were similar in terms of age, secondary operation incidence, and sex because of our selection criteria.

There were some differences and tendencies regarding operation time, analgesia duration, blood loss, complication rates, and excellent outcome ratings among Nuss, Ravitch, and Robicsek patients. The Ravitch procedure had a tendency to be longer than the Nuss or Robicsek procedure. There was a tendency for greater epidural and intravenous analgesia duration in the Nuss procedure (2.5 and 4.1 days respectively) compared to the Ravitch procedure (0.6 and 2.1 days respectively). The Nuss (28.6 mL) had a slight difference in blood loss as compared to the Ravitch procedure (111 mL). The non-displacement complication rates for the Nuss procedure varied much more than for the Ravitch or Robicsek procedures, and there was a tendency for greater non-displacement complication rates for the Nuss and Robicsek procedures than for the Ravitch (averages: 38%, 36%, and 12.5% respectively). The self-resolving complication rates were similar for the Nuss and Ravitch procedures. The Nuss patients (85%, 73-90%) had slightly better excellent outcomes than Ravitch (76%, 68-78%) patients and clearly better excellent outcomes than Robicsek (54%, 38-68%) patients. The good-excellent outcomes, however, were similar across all three procedures (Nuss 95%, Ravitch 96%, Robicsek 89%).

All other characteristics were similar. There was no difference in LOS, percentage requiring reoperation, or bar/strut displacement rates– the averages clustered and ranges overlapped for all three procedures (Table 4). Several studies in the pediatric Nuss and Ravitch groups reported postoperative improvement of symptoms, lung function, and cardiac function [24,53,54,56]. One study in the Robicsek group reported an improvement in symptoms and no change in lung function [61].

### Comparison of nuss groups

Independent of age, Nuss patients had minimal differences. The pediatric and adult patients’ operation duration, blood loss, LOS, displacement rates, and percentage requiring reoperation were similar. Analgesia duration, outcome ratings, complications, and symptoms all showed tendencies or slight differences. The pediatric and adult durations of epidurals had overlapping ranges (0–3 days and 2.8-3 days respectively), but the pediatric average (2.5 days) was below the adult range and average (3 days). The outcomes amongst adults were more variable. The excellent outcomes were similar, but the good-excellent outcomes showed a slight difference. There was a tendency for more pediatric self-resolving complications, but the total complications were the same in the two groups. We found no report of improving adult lung function with the Nuss procedure, but found reports of improved long-term lung function in children [46-48]. Investigations in each group reported improved symptoms and cardiac function [29,41,42,47-49].

### Comparison Ravitch group

The pediatric and adult highly modified Ravitch procedure patients had minimal differences [5,14,54,65]. The operation duration, analgesia duration, complication rates, percentage requiring reoperation, and postoperative clinical improvement (i.e. symptoms, lung function, and cardiac function) were simlar. Postoperative LOS, outcome ratings, and strut displacement showed tendencies or differences. Pediatric patients showed a tendency to stay in the hospital longer postoperatively. The good-excellent outcomes were the same for pediatric and adult patients, but pediatric patients showed a tendency for more excellent outcomes. There was a clear difference between pediatric (6.4%) and adult (0.0%) strut displacement.

## Review

This study compares pediatric and adult patients across contemporary PEx treatments, distinguishing age-based differences, unlike any previously published investigation. A handful of studies in the literature compare the Ravitch and Nuss procedures independent of age. Two small cohort (n < 30) studies attempt to compare Nuss patients across age groups. To our knowledge, no study compares the Ravitch, Robicsek, or surgical implants across age groups. In the following, we discuss the two most relevant previously published comparative studies by Nasr and colleagues and Kim and colleagues with three goals in mind: to corroborate our results with pre-existing literature, to highlight the unique results of this study, and to demonstrate conflicting findings in need of further investigation [26,32].

Nasr and colleagues performed a meta-analysis comparing Ravitch and Nuss procedures [26]. They included nine comparative studies focusing on Nuss and Ravitch procedures [22-25,27-31]. They analyzed these studies based on complication rates, LOS, duration of surgery, time to ambulation, postoperative pain management, and patient satisfaction. All nine studies included at least one of these outcome measures. Nasr and colleagues found no difference in complication rates between the Nuss and Ravitch procedures, corroborating our results. However, Nasr and colleagues found no difference in outcome ratings between Ravitch and Nuss patients. While we found no difference in excellent outcome ratings, our unique age categorization demonstrated more good-excellent outcomes among the pediatric Nuss population compared with the adult Nuss population [66,67]. We predict this slight difference will disappear in the future because the Nuss procedure has only recently been used for adult patients. In time, surgeons will propose additional modifications and adopt new techniques as they gain experience performing this operation on the less flexible adult chest wall. Already, Park and colleagues reported very impressive outcomes in numerous adult patients with minor modifications [68].

With regards to the outcomes of the pediatric group, we found that Nuss pediatric patients have a slight advantage in excellent outcomes when compared to Ravitch pediatric patients. Kelly and colleagues attempted to compare Ravitch and Nuss pediatric patients, but failed to obtain adequate enrollment in the Ravitch group, which limited their statistical analysis and power [24]. Without additional corroborating evidence, we speculate that the placement of bars beneath the bone and cartilage as opposed to incisions through the bone and cartilage, allows for a more natural course of growth and development of the anterior chest wall in pediatric patients, leading to more excellent outcomes [3,34]. This finding suggests that pediatric patients will have greater potential benefit from the Nuss procedure than the Ravitch; however, this should continue to be actively investigated.

We found a difference in LOS between adult Nuss and Ravitch groups, where Nasr and colleagues identified no difference. Similarly, Nasr and colleagues were unable to adequately compare epidural use, where we noted a clear difference between adult Nuss and Ravitch patients. While this study did not address cost, the need for greater lengths of stay and pain management suggest that the Nuss procedure is more expensive. This is consistent with current publications comparing the cost of PEx treatments independent of age [69,70]. However, we postulate that the longer operative time of the Ravitch procedure may offset some of the costs. In the pediatric group, our study found no difference in length of stay and only a slight difference in epidural duration between pediatric Nuss and Ravitch patients, consistent with Nasr and colleagues. Importantly, Nasr and colleagues meta-analysis contained mostly pediatric patients. This suggests that the difference in cost identified in the literature is a result of adult patients, not pediatric patients.

Nasr and colleagues reported greater reoperation rate due to displacement in the Nuss procedure patients. We found no difference in the percentage of patients requiring reoperation or bar/strut displacement between pediatric Nuss and Ravitch patients, but found a clear difference in bar/strut displacement between adult Nuss and Ravitch patients. We did not account for all post-bar removal recurrence or bar/strut displacements because of the lack of long-term follow-up in many included studies [71]. This limitation may explain the difference in findings. Nevertheless, all our groups had similar follow-up time averages, although the range for Ravitch patients was much greater. This variation in follow-up duration was a limitation of our study; accordingly, we may underestimate our postoperative outcome measures: outcome ratings, non-displacement complications, bar/strut displacements, and percentage requiring reoperation.

Our results suggested a greater tendency for bar displacement in adults, perhaps reflective of the greater rigidity of the adult chest wall [34]. To date many modifications, which our study did not consider, have decreased bar displacement. These include using stabilizers, suture fixation, stronger bars (especially in adults), and placement techniques [35,40,68,72-75]. Consequently, we anticipate that bar displacement rates will become similar between pediatric and adult Nuss patients as adult surgical techniques are further honed and the Nuss procedure gains popularity in adults.

Kim and colleagues conducted a small retrospective cohort study comparing Nuss procedure patients across age groups, which provided another useful comparison [32]. This included 39 pediatric patients, which they divided into <12 (n = 27) and 12–19 (n = 12) groups, and twelve adult patients (>19). They followed patients for an average of 3.4 years (1.7-4.3). They found that complications differed significantly between patients <12 and patients >12, but that complication rates were the same between patients 12–19 and >19. We initially intended to differentiate between pediatric patients (<12) and adolescents (12–19), but only found two studies that stratified their data appropriately, so we were obliged to combine pediatric and adolescent subgroups [32,33]. We had wanted to use 19 years of age as our cut-off between the pediatric and adult populations because significant developmental changes occur in the chest wall around this age; however, upon reviewing the available literature we had to modify our inclusion criteria [34]. Unlike Kim and colleagues, we found no difference, only tendencies, between adult and pediatric Nuss patient complication rates. In addition, we found no difference in operation duration or the percentage of patients requiring reoperation, where Kim and colleagues noted significant differences. We attribute these three differences to three factors – follow-up duration, cohort size, and surgical experience.

Kim and colleagues followed their patients for 3.4 years on average, while our studies averaged 2.1 and 2.0 years for pediatric and adult studies, respectively. Typically, bar removal occurs around two-years postoperatively for Nuss patients. Less than half (40.2%) of our Nuss patients had their bars removed. As a result, we underestimated the complication rate for both age groups. The complication rates that Kim and colleagues reported for their adolescent (58.3%) and adult patients (58.3%), however, were much higher than the average complication rates that we found (27.1%), even including bar displacement (as they did). Perhaps their small patient cohort contributed to this elevated complication rate. We only found comparable adult complication rates this high in other small cohort (n < 20) studies [32,36,41]. Another confounder may be surgical experience. Surgeons conducting these small cohort studies may not have much experience with adult PEx repair, potentially leading to higher complication rates. Nonetheless, there have been few reports of life-threatening complications in the literature [76,77].

We did not find a study that satisfactorily compared pediatric and adult Ravitch patients. In fact, we had much more difficulty than anticipated in finding Ravitch studies that met our age stratification inclusion criteria. We broadened our criteria to include more studies, but may have compromised the internal validity of our study. Most Ravitch studies included a very wide age range and, as a result, could not be included [9,23,28,65,69,78-90]. The only clear difference that we found between pediatric and adult Ravitch patients was strut displacement. The strut is typically attached to the ribs of the lower anterior chest posterior to the sternum with wire [81]. We speculate that strut displacement occurs more often in children because the ribs, to which the strut is attached, grow in the pediatric chest wall and are less developed, more malleable, and less calcified than in adult patients [2,3,34,88,91]. This suggests that the Ravitch is better suited for adult patients.

The results reported in this study should be considered for most PEx patients; however, there are limitations. The majority of our data came from retrospective cohort studies and we found no randomized investigations, which provide a higher level of evidence. Additionally, the follow-up time was minimal in some included investigations, which may underrepresent the long-term complications, outcomes, and reoperations; however, these provided significant information for the majority of the outcome measures assessed (operation duration, analgesia duration, blood loss, and length of stay). The reports of outcomes were largely based on subjective, though previously published, qualitative ratings (e.g. good-excellent, excellent), which may limit the reproducibility and consistency across patients and providers [63]. We did not consider procedural modifications, such as bar fixation techniques, or surgical collection criteria, such as patients with extreme asymmetrical pectus deformities, which may be susceptible to even higher complication rates with the Nuss procedure [80]. We did not examine patient satisfaction, which theoretically may favor the Nuss procedures because of smaller wounds/scars; however, in practice, Nasr et al. did not find significant difference in patient satisfaction [26]. We also did not include large populations of patients with extreme comorbidities, such as Marfans or scoliosis, which may need more careful consideration when choosing an appropriate treatment. Regardless, we performed an extensive search of the published literature, yielding large number of patients in our most essential groups (Nuss and Ravitch). We recognize that a meta-analysis would provide a more rigorous statistical comparison of the data we collected; however, the details to complete such a study are not available in the literature. Thus, we performed a highly systematic review, which provides a thorough, albeit less statistical, comparison of current PEx treatments. In doing so, this study attempts to address the question about the age appropriateness of different PEx treatments – namely the Nuss procedure and highly modified Ravitch procedure.

## Conclusion

Our analysis would suggest that completely asymptomatic PEx patients do not need to undergo operative treatment at an earlier age in order to achieve a better outcome. While many surgeons suggest that operating on children and adolescents results in better outcomes, the literature fails to corroborate this. Many studies do, however, support the observation that the Nuss procedure is easier to perform in younger patients, the Nuss and Ravitch procedures are safe and effective for pediatric and adult patients. There is no clear difference in outcome ratings between the Nuss and Ravitch populations across all age groups, but our results suggest slightly better outcomes in the Nuss pediatric group as compared to all other groups. Based on the recent increase in adult Nuss procedures and ongoing technical modifications, we recommend that adult outcomes continue to be monitored to see if this slight difference is just an artifact of surgical inexperience. On the other hand, we recognize that the Ravitch procedure has been around for decades, but still has slightly worse outcomes among pediatric patients. We recommend that uncomplicated pediatric patients with symptomatic PEx, therefore, receive the Nuss procedure. Finally, while there is not enough data in the literature to adequately compare alternative treatments, such as the Robicsek and surgical implant, current publications do not suggest any postoperative advantages; we recommend, therefore, that these procedures be avoided until the literature suggests that they are equally effective as compared to the Nuss or Ravitch procedures.

In the future, more rigorous long-term studies are needed, especially with regard to adults and the Nuss procedure. This will help to further evaluate the usefulness and appropriateness of each procedure. In addition, as new procedures are developed, such as the vacuum bell and mini-magnetic mover, they need to be compared with the Ravitch and Nuss procedures, which have become the accepted standard for PEx treatment. Until then, however, the literature supports the fact that there are viable treatments for patients of all ages.

## Abbreviations

LOS: Length of stay; PEx: Pectus excavatum.

## Competing interests

The authors declare that they have no competing interests.

## Authors’ contributions

WRJ made substantial contributions to conception and design; acquisition data, analysis, and interpretation of data; drafting and revision of this manuscript. DF made substantial contributions to the conception and design and revision of this manuscript. SS made substantial contributions to the conception, analysis and interpretation of the data, and the revision and final approval of the version to be published. All authors read and approved the final manuscript.
